# Provider’s Perceptions of Barriers and Facilitators for Latinas to Participate in Genetic Cancer Risk Assessment for Hereditary Breast and Ovarian Cancer

**DOI:** 10.3390/healthcare6030116

**Published:** 2018-09-17

**Authors:** Alejandra Hurtado-de-Mendoza, Kristi Graves, Sara Gómez-Trillos, Lyndsay Anderson, Claudia Campos, Chalanda Evans, Selma Stearns, Qi Zhu, Nathaly Gonzalez, Vanessa B. Sheppard

**Affiliations:** 1 Department of Oncology, Georgetown University Medical Center, 3300 Whitehaven Street, Suite 4100, Washington, DC 20007, USA; kdg9@georgetown.edu (K.G.); sg1328@georgetown.edu (S.G.-T.); ss4081@georgetown.edu (S.S.); qz66@georgetown.edu (Q.Z.); 2 Department of Nursing, California State University, Sacramento, CA 95819-6096, USA; ltw7@georgetown.edu; 3 Nueva Vida, DC Office—Alexandria, 206 N. Washington St. Suite 300, Alexandria, VA 22314, USA; ccampos@nueva-vida.org; 4 Penn Medicine Nudge Unit, University of Pennsylvania, Philadelphia, PA 19104, USA; chaevansc3@gmail.com; 5 Capital Breast Cancer Center, 1000 New Jersey Ave, SE, Washington, DC 20003, USA; ng472@georgetown.edu; 6 Department of Health Behavior Policy, Virginia Commonwealth University, Richmond, VA 23219, USA; Vanessa.Sheppard@vcuhealth.org

**Keywords:** hereditary breast and ovarian cancer, genetic counseling, genetic testing, Latinas, Provider’s perspectives, barriers

## Abstract

The Comprehensive Cancer Network (NCCN) recommends genetic cancer risk assessment (GCRA) referral to women at high risk of hereditary breast and ovarian cancer. Latinas affected by breast cancer have the second highest prevalence of *BRCA1/2* mutations after Ashkenazi Jews. Compared to non-Hispanic Whites, Latinas have lower GCRA uptake. While some studies have identified barriers for GCRA use in this population, few studies have focused on health care providers’ perspectives. The purpose of the study was to examine providers’ perceptions of barriers and facilitators for at-risk Latina women to participate in GCRA and their experiences providing services to this population. We conducted semi-structured interviews with 20 healthcare providers (e.g., genetic counselors, patient navigators) recruited nationally through snowballing. Interviews were transcribed. Two coders independently coded each interview and then met to reconcile the codes using Consensual Qualitative Research guidelines. Providers identified several facilitators for GCRA uptake (e.g., family, treatment/prevention decisions) and barriers (e.g., cost, referrals, awareness, stigma). Genetic counselors described important aspects to consider when working with at-risk Latina including language barriers, obtaining accurate family histories, family communication, and testing relatives who live outside the US. Findings from this study can inform future interventions to enhance uptake and quality of GCRA in at-risk Latina women to reduce disparities.

## 1. Introduction

*BRCA1/2* are the most commonly identified genetic mutations responsible for hereditary breast and ovarian cancers (HBOC) [1]. For the purposes of this paper, HBOC represents an inclusive term, which includes other genes that increase the susceptibility for breast or ovarian cancer. *BRCA1/2* carriers have 50–80% and 15–40% lifetime risks of developing breast and ovarian cancer, respectively [2]. Breast cancer survivors with *BRCA1/2* mutations are three times more likely to develop contralateral breast cancer than non-carriers [3]. The National Comprehensive Cancer Network (NCCN) recommends Genetic Cancer Risk Assessment (GCRA) referral to women at high risk based on personal and/or family history of cancer [4]. GCRA includes genetic counseling plus the option of single or multiplex panel genetic testing. A positive test result impacts treatment decisions for newly diagnosed patients [5] and uptake of risk reducing surgeries [6]. Risk-reducing prophylactic surgeries can reduce breast cancer risk by ≥90% [7,8,9] ovarian cancer risk by 85–90%, [10,11] and increase life expectancy among mutation carriers [12]. Additionally, there are therapies targeting *BRCA1/2* carriers with metastatic cancer that are more effective than standard therapy [13,14].

Latinas affected by breast cancer have the second highest prevalence of *BRCA1/2* mutations following women of Ashkenazi Jewish ancestry [15]. The prevalence and spectrum of *BRCA1/2* deleterious mutations vary across Latin American subpopulations [16]. Despite the national priorities to increase racial and ethnic diversity of patients who participate in GCRA [17] striking disparities remain. Compared to non-Hispanic Whites, Latinos have lower awareness of GCRA [18], lower breast cancer genetics knowledge [19,20,21], lower health literacy and numeracy [22,23], and lower testing uptake [24,25].

Latinas report positive attitudes and interest in participating in GCRA [26,27,28,29]. Barriers to GCRA uptake in this population include access, language, and psychosocial factors (e.g., awareness, emotional concerns, competing priorities) [20,26,28,29,30,31]. Additionally, population-based data showed that minority breast cancer survivors are more likely to report unmet needs for genetic testing discussions. Although Spanish-speaking Latinas had the strongest desire for testing, Latinas were almost five times more likely to have unmet needs of discussion about testing compared to non-Hispanic Whites [32]. Underserved populations seen in community settings are less likely to receive referrals to genetic counseling [33]. Although there is mixed evidence [27], some studies have shown high uptake of GCRA when appropriate referrals are made [34] and when barriers to testing are reduced [35].

Even when Latinas participate in counseling, effective communication during counseling can be compromised due to language, cultural differences, or communication mode (face-to-face vs. telephone). Few genetic counselors in the US speak Spanish [36]. When medical interpreters are used in genetic counseling sessions, there is a great risk of miscommunication [37,38,39].

Prior studies have focused on at-risk Latinas [20,28,29,30] or conducted direct observation of counseling sessions [37,38,39]. Little is known about the perspectives of different types of healthcare providers that play a key role in navigating and providing counseling services. The goals of the present study were to examine providers’ perceptions of barriers and facilitators for at-risk Latina women to participate in GCRA. We sought to describe providers’ experiences, including challenges and strategies to overcome those challenges, in providing services to this population. 

## 2. Methods

### 2.1. Participants and Procedures

We recruited providers through postings on list-serves and snowballing. Providers included genetic counselors (*n* = 11), geneticist (*n* = 1), patient navigators (*n* = 3), social workers (*n* = 1), psychologists/counselors (*n* = 2), doctors (*n* = 1), and nurses (*n* = 1). Most (90%) were female. Most worked on academic hospitals (*n* = 12), followed by hospitals/medical practices (*n* = 4), and community clinics/community-based organizations (*n* = 4). The research team reached out to schedule interviews, conducted by phone or in person, at a time convenient for the providers. All participants signed informed consents. Participants received a $10 gift card. The study protocol was approved by Georgetown University Institutional Review Board (The IRB number is 2016-0193 and the approval dates were: 10 March 2016 to 9 March 2017). 

### 2.2. Interview Guide

The semi-structured interview contained a set of core questions and questions that varied depending on the role of the provider. Providers were asked about their experiences working with Latina women, the services they provide to Latina women, whether and how they assess the risk of HBOC in this population, and barriers and facilitators for Latinas to use genetic counseling and testing services. Navigators and social workers answered more specific questions about the process of navigating women into counseling and testing services and challenges that both at-risk Latina and providers face. Genetic counselors were asked to discuss their experiences providing counseling to Latina women, common misconceptions about HBOC and GCRA, challenges they face while counseling as well as strategies they use to overcome those challenges.

### 2.3. Qualitative Analysis

Interviews were recorded and transcribed verbatim. Four interviews were conducted in Spanish and sixteen in English. Two authors did an initial read of four interviews and independently developed a list of codes emerging from the transcripts. Then, they reconciled their codebooks to develop a master codebook, including definitions of each code. Throughout the entire coding process, the codebook was adjusted and refined. Using the Consensual Qualitative Research Framework [40], three authors independently coded the interviews in the language they were conducted (two authors per each interview) in the qualitative analysis software (Dedoose) [41], then met to reconcile any disagreements in coding. We then selected quotes representative of themes. 

## 3. Results

We used participants’ descriptions about the services they provide and their experiences working with Latina women (e.g., number of Latino clients) to classify providers’ experiences with Latinos as low (e.g., only see few Latinos), intermediate (e.g., see some Latino clients), and high (a large proportion of their clients are Latinos). Five providers saw a small proportion of at-risk Latinas, three had an intermediate experience, and twelve had a high experience working with Latinos. 

### 3.1. Perceived Facilitators to Participate in GCRA

Providers identified facilitators for at-risk Latina to participate in GCRA included informing the risk of family members, informing treatment and prevention decisions, and following doctor’s recommendations (see Table 1 for additional quotes).

**Family**. Providers consistently mentioned that family is very important among Latinos. Thus, trying to prevent cancer in relatives was a primary motivation for Latina women to participate in GCRA.

“I think family is really at the forefront of a lot of the decision making for Latina women. Family, that is the first thing that is brought up (…) they have more of a collective mentality”

—Genetic counselor

**Informing treatment and prevention decisions.** Providers mentioned that another motivation for women to participate in GCRA is to understand their individual risk for a second cancer and evaluate the different prevention and treatment options.

“If they are already diagnosed with cancer [women seek genetic testing] to know whether or not they should have done a bilateral mastectomy”

—Navigator

**Doctor recommendations (Respect).** Several providers noted that at-risk Latinas tend to use the GCRA services when doctors recommend it. Even if the purpose of the visit may not be sufficiently clear, providers shared that often out of respect (cultural value of *respeto*), Latinas try to follow medical recommendations without questions.

“People are sent because their doctors go “you are 35 and you need to go to genetic counseling” and you get people who come here and go “I’m here because my doctor told me I had to, but I don’t know”

—Genetic counselor

### 3.2. Perceived Barriers to Participate in GCRA

Providers identified barriers for at-risk Latina to participate in GCRA included cost and insurance, referrals, awareness, education, and language, logistic barriers, emotions, spirituality, fatalism, stigma, and systemic barriers (see Table 1 for additional quotes).

**Cost and Insurance.** Most providers, especially navigators/social workers, highlighted high costs and lack of insurance as one of the main barriers for Latinas to participate in GCRA. Some were not very familiar with the labs assistance programs or insurance coverage. Of the four navigators/social workers, one worked at a setting where both counseling and testing were available, two worked a setting where only testing was available, and one worked at a setting where neither was available.

“The cost can be prohibitive…I don’t know what percentage insurance covers genetic testing”

—Navigator

Navigators and social workers described loopholes in some State Assistance Programs that had limited coverage (e.g., ovarian cancer cases not covered). They also noted challenges to obtaining authorization from the insurance companies that often resulted in delays. Navigators and social workers further identified challenges for finding insurance coverage for the needed screening and prophylactic surgical procedures following receipt of a positive test for a deleterious mutation. 

One provider described how the limited access to testing might lead to suboptimal surgical decisions: “A surgeon may possible recommend to them (Latinas) you might want to think about a bilateral mastectomy just based on your family history and your risk factors, sometimes you don’t need a test… (...) it could really be a workaround but they’re not really getting the testing so we don’t know if we’re doing something that might not be (needed) (...) and it’s not helpful to the family member because there is no test done” (Nurse navigator)

In contrast to patient navigators and social workers, genetic counselors appeared to perceive that cost and insurance were no longer significant barriers. They noted that the cost of testing had decreased dramatically over the last 15 years. They also mentioned the increased coverage by insurance companies, noting the implementation of the Affordable Care Act, the availability of assistance programs in some states, and other available funds from hospitals, foundations, or grants. 

Genetic counselors emphasized the importance of informing at-risk women about the existing resources to cover the costs of testing, since women may be unaware.

“The other problem is the payment, people think that the insurance will not cover it and they are convinced that they will have to pay for it when in reality, for most people, insurance will cover it”

—Geneticist

However, some providers mentioned greater challenges with covering the cost of genetic counseling for uninsured women than covering costs of testing. To overcome this barrier, some counselors used grant funds to cover the counseling, while others provided free services.

“Most laboratories have testing hardship program[s], so if someone is low income and we can get them free testing setup, that is actually quite easy, but it is kind of ironic that the genetic counseling part can sometimes be a lot harder to get because there aren’t that many free resources for genetic counseling”

—Genetic counselor

**Referrals.** Providers described having referral procedures in place at their institutions and also getting some referrals from community-based organizations. However, they also noted gaps and limitations in the referral process. Reasons cited for the suboptimal referral included doctors’ limited knowledge about HBOC risk factors and referral guidelines, primary care doctors’ not assessing or addressing family history of cancer, or patients’ low awareness and knowledge about HBOC and GRCA services. Providers’ limited knowledge about financial assistance programs also resulted in uninsured patients not being referred to GCRA under the assumption that they will not be able to cover the costs.

“The providers may not always guarantee that the testing will be covered. A lot of my patients are told that it’s not going to be covered when it actually is, so they are never referred to start”

—Genetic counselor

Additionally, due to the difficulty of covering counseling, some patients get testing without prior counseling.

“These are all staged breast cancer patients who have already been identified as breast cancer patients and they had been recommended for testing and counseling. Sometimes they get the testing but they won’t get the counseling (because extra cost)”

—Navigator

**Awareness, Education, and Language.** Providers noted that one of the main barriers is the lack of awareness about genetic counseling and testing services in the Latino community. Limited awareness about the services is also exacerbated by language barriers, the limited availability of informational resources in Spanish, and low familiarity with genetics. “Latina women may think ‘how come am I going to go to the doctor if the doctor will not understand what I say or I will not understand what the doctor says’” (Geneticist)

**Logistic barriers** included transportation, childcare, having appointments during business hours, and the subsequent difficulties getting off work. Providers mentioned strategies to address barriers such as offering telephone counseling, covering transportation costs, or trying to schedule multiple visits to the health care team on the same day.

“A lot of people work full time and have a lot of problems getting to the appointments during business hours, or have jobs where they can’t leave work or they would have to take time off, they can’t afford the day off or they employer won’t let them take the day off”

—Genetic counselor

**Emotions.** Providers mentioned that HBOC can elicit emotions like fear, worry, and guilt which may hinder GCRA participation. Additionally, for newly diagnosed patients, participating in GCRA can feel overwhelming while they are in active treatment and processing emotionally the cancer diagnosis. “A lot of times it is kind of scary for all of them, especially the patients that aren’t breast cancer patients finding out whether or not you have this gene and then having to worry about children or other family members can be scary” (Navigator)

**Spirituality.** Providers noted that spirituality and faith is very important in the Latino community, as many believe that their health outcomes are “up to God”. Faith is often brought up as a source of strength. However, in some cases, spirituality may hinder participation in treatment. “I am not going to do the treatment because God is going to decide if I live or don’t live from this” (Psychologist/counselor)

**Fatalistic beliefs.** Providers also noted that for many women cancer is synonymous to death or that having cancer is inevitable or not under their control. “I have people that come in that are just convinced they are going to get cancer, like yeah, that is just the way it is. Everyone gets cancer; it is not if, it is when” (Genetic counselor)

Providers mentioned that some people are not ready to take action by undergoing testing, some people do not want to know if they are going to have cancer or not, or do not want to hear about management options.

“Some people are not ready in that moment and prefer to wait, especially if they had a negative experience with a relative, so there is people that say I will die of something so I prefer not to know”

—Navigator

**Stigma.** Providers reported that cancer is often stigmatized in Latino communities, especially in certain subgroups like older generations, women from rural areas, women with lower education, and women recently arrived in the US. 

The use of the phrase ‘genetic counseling’ can carry stigma, as it can be misunderstood as psychological counseling and the idea of being ‘crazy’. Providers shared that some of their Latino patients believe that just saying the word cancer increases the chances of developing cancer. Providers reported that often people do not want to share the cancer diagnosis with others for fear of being gossiped about or ostracized.

“I remember one patient in particular from Bolivia and she felt much safer being here and talking about it with people. She said that if she were to bring this up back home that people would be doing a lot of gossiping and that it would be malicious and hurtful and it would be detrimental to her in various ways”

—Psychologist/counselor

Additionally, providers described that breast and ovarian cancers can be stigmatized since they can pose a threat to femininity. Other sources of stigma included the belief that women got cancer because they did something wrong (and that God is punishing them) and thinking that cancer is contagious. 

Providers noted that this stigma often resulted in the lack of family communication about cancer and not sharing the cancer diagnosis, which could decrease awareness about cancer in the family.

**Systemic Barriers.** A few providers mentioned other potential barriers including fear of discrimination, medical mistrust (especially in relation to the coverage), difficulties navigating the medical system or not being familiar with a medical system that has specialists (rather than a general doctor), competing priorities, and immigration status.

“Sometimes, in a few instances, some people are afraid to fill out paperwork for the free testing because they were undocumented. But I would have said this is fairly rare.”

—GC

### 3.3. Genetic Counselors’ Experiences during Counseling

The genetic counselors and the geneticist identified several aspects that are important to consider when providing counseling to at-risk Latina women including language, education and misconceptions, gathering family history, communication with relatives, testing relatives outside the US, logistics, and engagement in decision-making/respect (see Table 2 for additional quotes).

**Language.** Given the dearth of Spanish-speaking counselors, language was noted as one of the biggest challenges. The three counselors/geneticist who spoke Spanish (out of 12) highlighted how important it was for Latinas to get counseling in Spanish. 

Most monolingual (English) genetic counselors use interpreters who participate by telephone or in person. While counselors valued the availability of interpretation services to provide services to clients with limited English proficiency, they also noted several challenges and shortcomings. For example, using interpreters can hinder building rapport between the counselor and the patient, add time to the length of the session, and, when using telephone interpreters, make it harder for the counselor to get feedback from nonverbal cues.

“(The sessions with interpreters are) about a third longer because you have to repeat everything twice (…) a lot of what we do is try to build rapport with the patient and that’s just an added layer. I have had good sessions with interpreters but it does make it a little more difficult if a patient is more engaged with the interpreter, they [the patients] are looking at the telephone, and it’s hard to make the contact with them. These [are] little things and nuances in a session but overall might really affect the communication.”

—Genetic counselor

Genetic counselors noted that while in-person interpreters might be preferable, it is still challenging to ensure that the right message is delivered. For instance, interpreters appear to summarize and cut off important information and may translate key terms into Spanish words that have different meanings. 

In some cases, interpreters might completely mistranslate the information because they become emotionally involved and they do not want to worry patients or because they are unfamiliar with the nuances of genetics: “Some things were more serious than others. For example, a positive test result would occur and the translator would say ‘you have cancer’”. (Genetic counselor)

**Education and Misconceptions.** A number of genetic counselors shared that many of their Latina patients, especially patients who migrated from rural areas in Latin America or those with low levels of formal education, did not have enough knowledge to understand some of the basic genetic concepts. Moreover, some counselors noted the availability of Spanish-language informational brochures, but emphasized that the language was too technical to be useful to the patients. One counselor suggested that providing too much scientific information might not always be necessary. Instead, she recommended providing a brochure that summarizes the key points and after explaining these points in simple terms, spending more time considering and addressing the patient’s actual worries (e.g., talking about the risk to daughters instead of *BRCA1* being a tumor suppressor). The counselor suggested that this approach leaves open the possibility of further describing the scientific nuances based on patient preferences. 

Some of the most frequent misconceptions that counselors reported they encountered during sessions revolved around genetic counseling/testing and biological concepts (cancer, genes, and heredity). 

*Genetic counseling/testing*: Genetic counselors noted that many people associate genetic counseling with psychological counseling. Other prevalent misconceptions around GCRA included: (1) Genetic testing will provide a diagnosis for cancer, (2) genetic counseling will inevitably lead to genetic testing, (3) a positive result means patients will have to do prophylactic surgery, (4) a negative result means that patients do not have to worry anymore, and (5) the test results can change.

“That’s a question I get a lot. “You know, well, in a few years if you did this test again, could I get back something different?”

—Genetic counselor

*Cancer, genes, and heredity concepts:* Counselors identified a big gap in patients’ understanding about the different causes of cancer. They mentioned that some Latina patients believe that they caused the cancer (e.g., stress, bad diet) or that all cancers are hereditary (e.g., everyone has cancer cells waiting to be turned on). Counselors observed a general lack of knowledge that gene alterations can also be passed by and to male relatives.

“One of the major misconceptions is that these breast cancer genes can only come from the mom’s side of the family (…) So that is something that comes as a shock to a lot of people, especially when you’re doing the family history right in front of them (…) and you start to point out things in the dad’s side of the family”

—Genetic counselor

Genetic counselors identified that patients appear to greatly misunderstand patterns of inheritance: “Sometimes people also think (…) “I look just like my mom and she had cancer, and because I act just like her (…), so I must have it too.” (Genetic counselor)

Lastly, providers discussed how different types of cancer such as ovarian, cervical, and uterine cancers are confused or broadly referred to as “female cancers”.

**Emotions**. Counselors highlighted that emotions are an important aspect of genetic counseling. For instance, counselors noted that some Latino patients feel that genetic information is too much to process and fear the results.

In contrast, other patients feel relieved by the results (even if test is positive) because it puts an end to uncertainty and patients feel that they finally have a starting point to do something about their cancer risk. Counselors mentioned that some women appear to feel guilty because they think that they did something to cause the cancer or that they could have passed a mutation to their children. For example, counselors commented that grandmothers reflect guilt because they might have “passed it to future generations”, mothers show concern for their children and feel guilty, and fathers feel guilt and sadness as they are protective of their daughters. Additionally, counselors reported that young women who may not yet have children may feel shocked when faced with the possibility of removing their ovaries. 

Several counselors emphasized the importance of normalizing people’s beliefs about genetic mutations (e.g., many people have mutations), emphasizing that people cannot control which genes they pass, acknowledging and legitimizing patients’ feelings of guilt and reframing receipt of a genetic test result as important information they are providing to their family that can allow them to prevent cancer.

“I want my patients to see that information as a gift they provide to their kids. The perception is ‘Oh, I am causing cancer to my kids.’ I try to change that perception to ‘this is random (…) try to see this as a present to your daughter or son, you are giving them the possibility of being proactive instead of reactive in their healthcare’”.

—Genetic counselor

**Family History.** Genetic counselors indicated that gathering accurate family history can be challenging since Latina women can lose contact with some relatives due to geographic distance or because they were raised by only one side of the family. Thus, it can be hard to know the vital status of some relatives. Additionally, counselors commented that women may not know the cause of their relatives’ death due to the stigma around cancer, which is common in older generations, or the limited access to doctors, particularly in rural areas. Counselors suggested that sometimes relatives ask doctors not to disclose the diagnosis to the person diagnosed with cancer to avoid worrying them. Additionally, providers noted that due to the violence and political conflicts in several Latin American countries, many relatives die young, so it is impossible to know if they would have developed cancer at a later age.

“There is like this hole certainly in the past, you know, this old taboo that people don’t talk about cancer. And so we did not know what happened to grandma, so and so, or grandpa, so and so, or uncle so and so or whoever was, they just got sick and then they died and we really don’t know what happened”

—Genetic counselor

Even when women are aware of a cancer diagnosis in the family, they may not have accurate information about which type of cancer it was since they often confuse cervical, uterine, and ovarian, or just refer to them as “female cancers”. Counselors reported difficulty in obtaining medical records to verify the accuracy of self-report. Although having an incomplete family history is a limitation, fortunately it has become less important due to the ability of doing multiple panel testing and the reduction of testing costs. 

**Communication with relatives.** Counselors indicated that they try to encourage patients to communicate with relatives, including sharing the test results. Some counselors noted that while some parents embrace testing in the family, other families have a hard time encouraging relatives to do the test for several reasons including feeling guilty, having lost contact with some relatives, not even sharing the cancer diagnosis to avoid worrying others or being a burden to the family, or to avoid showing a sign of weakness to the family. 

Some counselors voiced their challenges tracking whether women shared the information with relatives, especially when relatives were living outside the US.

“I usually pass it along to my patient and hope that they pass it on to their relative. But yeah, it can be difficult and frustrating because either from just the location or just the relatives not communicating well, it’s hard to get information to the family”

—Genetic counselor

A counselor explained that while writing letters in English and Spanish for relatives was important, it was not always enough if there was no follow-up. Once she had a client diagnosed with breast cancer, who was the sister of one of her clients who had tested positive, yet the newly diagnosed patient was unaware of this information. This case led her to modify her practice to spend more time discussing communication issues and trying to address barriers for testing for the relatives. Another counselor was involved in an intervention that included facilitating communicating results to relatives that participants found extremely useful.

**Testing Relatives Outside the US.** Several counselors mentioned the importance of taking into account the implications of testing for relatives who live outside the US. For instance, women could feel unease if they think that the test information may not benefit their relatives who live in Latin America and may not have access to the services.

“Something that is more unique to the Latina population I see, at least compared to other of my patients, a lot of their relatives are not here in the United States. So that brings up questions if I have this information will that benefit my relatives? Will they be able to get tested? Will they be able to get the screening they need or will they be able to get the information they need?”

—Genetic counselor

Although facilitating testing for relatives outside the US was challenging, counselors shared different strategies they use. Strategies included identifying services in other countries (typically limited and not existing in every country), testing relatives when they come to the US, sending testing kits with their clients when they travel, asking the relatives to order the test online, tapping into a research registry with collaborators from Latin America, or posting in the National Society of Genetic Counselors website to find if anyone knows about resources in different countries. 

**Logistics.** Difficulty reaching out patients by phone was mentioned as a logistic challenge.

“Sometimes it’s just really hard for me to get back in touch with the patient. Sometimes their phone numbers won’t work or they’ll have voicemail and I’ll call with the translator and I’ll call and leave a message but I don’t know if they’ve checked their message, there is just those like logistical things.”

—Genetic counselor

**Engagement in Decision-Making/Respect.** Some counselors reported that in general Latinas tend to be less engaged in decision-making and be more compliant with medical recommendations.

“I feel like maybe there are fewer questions. And so sometimes I wonder, “is it something getting lost in translation? Do they not feel comfortable?” It’s hard because you want to make sure that everyone has the information to make an informed decision. But there is very little push back (…)”

—Genetic counselor

One counselor explained that the lower engagement in decision-making might be due to the paternalistic approach in medicine that is common in Latin America. For Latina women, the non-directiveness mandate of genetic counseling can be confusing. One recommendation was to use a more directive approach while always stressing that the ultimate decision is theirs.

“When we ask them ‘would you like to do the test or not?’ the patients are confused and tell me ‘well, you are the health professional, you should decide whether I do the test or not.’ (…). We try to be a little more directive and tell them ‘In my professional opinion I recommend this test (…) but ultimately the decision is yours.’”

—Genetic counselor

## 4. Discussion

To our knowledge, this study is among the first studies to assess providers’ perspectives about GCRA in at-risk Latina women [42]. Results related to motivators and barriers to participation in GRCA among Latinas confirm and extend prior evidence from research conducted with at-risk Latina patients. Reinforcing findings reported from studies with Latina patients, motivators to participate in GCRA included informing family risk, informing treatment/prevention decisions, and following doctors’ recommendations. Also consistent with prior work was providers’ identification of barriers related to included cost/insurance, suboptimal referrals, awareness, language, education, and stigma [20,26,29,43,44,45]. Below we identify where the present study findings expand prior work. 

Our results support previously identified cost and insurance barriers to GRCA among high-risk Latinas [26,29,45]. A relatively unexplored barrier we identified was the lack of familiarity of some providers (e.g., patient navigators and social workers) with laboratory assistance programs. Fostering communication between genetic counselors and navigators/social workers within and between institutions would be helpful to reduce the gaps in the referral process and to increase awareness about the reduced cost of testing and the availability of assistance programs. While increasing awareness about assistance programs may enhance the uptake of testing, access to genetic counseling could still be challenging since commercial genetic testing laboratories’ assistance programs are currently designed to provide coverage for testing only. Thus, efforts to enhance underserved populations’ access to genetic counseling are needed. Other access barriers beyond initial GCRA, such as referral and access to recommended screening and risk reduction surgeries, will also need to be addressed to ensure that recommendations for risk management are followed. Efforts that target training to patient navigators and community health educators about how to identify and refer high risk Latinas to genetic services may help bridge the gap between community members and genetic specialists. One such program is called “Árboles Familiares” (Genealogical Trees). This program aims to train community health workers, patient navigators and promotors to assess the risk of HBOC and provide appropriate referrals to at-risk Latina women [46]. 

The present findings also suggest that clinical guidelines to refer patients to genetic counseling prior to genetic testing are not always followed, in part due to the difficulty of covering costs of counseling. These observations by providers complement results in a recent study by Katz and colleagues [47] that identified important gaps in counseling uptake among at-risk breast cancer survivors: One-quarter of survivors did not receive any type of counseling, 31% received physician directed counseling, and only 43% had formal counseling. Thus, there is a need to further understand the low rates of genetic counseling uptake as well as the referral process in different settings (e.g., who makes the referral to counseling, which provider orders the test, etc.) and with heterogeneous populations to understand why the guidelines are not met. Evidence from community samples suggest that Latinas may be less likely to receive counseling. In a small study that included 20 at-risk Latina breast cancer survivors recruited from community settings, only 35% had testing and none received counseling [44]. Future research can examine outcomes among patients with and without genetic counseling and also explore if alternative genetic counseling delivery models, such as streamlined approaches to pre-test genetic education [48] may yield benefits in terms of reach and efficiency. 

Our results reinforce emerging evidence and contribute novel information in terms of factors that relate to the quality of genetic counseling for at-risk Latinas. Consistent with the seminal work of Joseph [37,38,39], we found that language and use of interpreters represented major barriers to provision of genetic counseling for at-risk Latinas. Counselors in the present study noted the impact of interpreters on logistics (extra time), relationship building (e.g., reduced rapport), and content (reduced information accuracy). Current efforts to train interpreters about HBOC can be important to increase the accuracy of interpretation during genetic counseling [49]. Additionally, diversifying the workforce to increase the number of Spanish-speaking Latino counselors will be important [50], as well as exploring alternatives such as Telephone Genetic Counseling with Spanish-speaking counselors [51]. 

Beyond language barriers, counselors highlighted other communication issues, including Latinos’ lower engagement in decision-making and potential confusion with the non-directive approach typical in genetic counseling. These findings support prior studies that examined genetic counseling in Latinos [37,39,52,53]. Although non-directiveness is a professional mandate of genetic counseling to preserve patients’ autonomy [53], it may be problematic for certain patient groups. A sociolinguistic analysis of prenatal genetic counseling showed that counselors’ use of indirect speech to avoid being directive could be confusing for patients, as they need to infer the recommendations and their particular concerns were not addressed [54]. This problem may be exacerbated for Latinos since they may be accustomed to more prescriptive approach from medical providers and expect clear, actionable recommendations. Thus, counselors may need to adapt their communication style to offer more clear advice while encouraging patient’s autonomy [53] and/or to explain to patients about the shared decision-making model [39]. Cultural competence training programs would be important for counselors to increase their ability to communicate effectively with diverse populations [55,56].

Counselors highlighted several factors that can affect family communication among high-risk Latino families. Our results complement findings from direct observation of counseling sessions with at-risk Latinas and Latina patients’ perceptions [26,37,38]. First, taboo and stigma about cancer decrease communication between family members. Second, when families are physically separated or have lost touch, patients are reluctant to call to talk about cancer. Third, feelings of guilt or not wanting to be a burden limit communication. Fourth, communication issues affect the gathering of complete and accurate family history. Additionally, older generations did not go to the doctors, so clients might not know the cause of death of their grandparents or the type of cancer. Finally, when it is necessary to test relatives outside of the U.S, it is very challenging to encourage clients to reach out and get the right message across. However, even if the relatives abroad want to have genetic counseling/testing, the services are often unavailable in their countries—especially if they live in rural areas. Family communication is key for capturing accurate family history, sharing the results, and enhancing cascade testing when needed to ensure that whole families can benefit from GCRA. Developing and culturally adapting training programs such as the KinFact [57] will be key to enhancing family communication among at-risk Latino families. These programs should address stigma and other specific challenges that hinder communication in this population (e.g., geographic distance, feelings of guilt, etc.). 

Another specific aspect to providing services to at-risk Latina, which is common with other immigrant populations, is the need to explore testing options for relatives outside the US, especially given the evidence that relatives of *BRCA1/2* mutation carriers who live outside the US are less likely to be tested [58]. This may be explained by the limited availability or the high cost of genetic testing for HBOC in most Latin American countries [59]. As many as 63% of Latino immigrants remain attached to their home country, traveling and communicating with relatives [60]. Given that a high percentage of Latinas in the US have family members living outside of the country, genetic service providers and researchers will need to build and systematize the current efforts that counselors are exploring to test relatives outside the US. These efforts can help maximize the benefits of GCRA—including cascade testing—and ensure that genetic counseling is responsive to the needs and concerns of the Latino population. 

This study had some limitations. We recruited a convenience sample of mostly female providers through list-serves and snowballing, so findings may not be generalizable to other providers who work with Latino communities. Additionally, we did not formally collect information about the percentage of at-risk Latinas that providers see. We had a small number of participants representing each profession and some were under represented (e.g., surgeons) or not represented (e.g., primary care providers). Future studies should include a greater number of providers from different professions and specialties. Despite the limitations, the study had several strengths. This study triangulates prior research that focused on at-risk Latinas and offers important insights from the provider’s perspective. The in-depth interviews with different types of providers yielded important insights into various aspects of GCRA services with at-risk Latinas. These findings can inform future interventions to enhance uptake and quality of GCRA in at-risk Latina women to reduce disparities in this underserved population. Finally, this study represents an important contribution to a scarce literature about providers’ experiences working with at-risk underserved populations [61]. 

## 5. Conclusions

This paper examined different provider’s perspectives about the facilitators and barriers for at-risk Latinas to participate in GCRA. Additionally, genetic counselors discussed their experiences providing genetic counseling to at-risk Latinas, including the challenges they face as well as the strategies they use to overcome those challenges. Findings from this study can inform the development of culturally targeted interventions to enhance the uptake and quality of GCRA in at-risk Latina women to reduce disparities.

## Figures and Tables

**Table 1:** Facilitators and Barriers to Participate in genetic cancer risk assessment (GCRA).

Facilitators	Family
	“I think that time and time again if you are thinking about Latina women specifically, their family has to be a big motivator. Common questions are: what is up with my daughter?” (Genetic counselor) “When they are doing the testing they are looking at a different perspective of being able to provide that new information for their family, so that the legacy of cancer does not continue in their family” (Genetic counselor)
	**Inform Treatment/Prevention Decisions**
	“Specifically women with ovarian cancer, it could help if they have a BRCA mutation, it could help with treatment options because there is a specific chemotherapy drug called “lemprasa”.” (Genetic counselor) “We know what caused the cancer, we can test another family member so that we can test: are they actually at risk for the same thing? And if they are, we are going to start taking care of them differently.” (Genetic counselor)
	**Doctors’ Recommendations**
	“Culturally, they tend to follow what the doctor is recommending” (Navigator) “What the doctors are telling them is what they should do. And they want to do everything that they can and they can make sure that they are very grateful (…) following up on whatever is recommended by them” (Genetic counselor)
**Barriers**	**Cost and Insurance**
	“I think the biggest barrier is getting them to take the test when they don’t have financial resources” (Navigator) “Finances are probably, potentially a barrier or a perceived barrier… Usually, nine times out of ten, we can find some way (to cover the test), whether it is going to a laboratory or coding it in the right way. We are at a level where people don’t have to pay anything for something that they can’t afford… In my experience, finances are becoming less of a barrier” (Genetic counselor)
	**Referrals**
	“Sometimes we have had some insured patients that have gone through counseling. But without insurance? It is out of the question. There is no mention of it… If there is a patient without insurance we can’t even give them the opportunity to talk about it, to explain about it” (Navigator) “We see very few Latinas referred to genetic counseling. I think they don’t know that they need to do it, maybe their doctors do not refer them or they don’t discuss about relatives with cancer. It is important to explain that if they have cancer in the family, they should talk with their doctor” (Geneticist)
	**Awareness, Education, and Language**
	“I think the bigger part of the barrier is that educational materials are not available in Spanish” (Navigator/social worker) “Lack of knowledge that these services exist, there is not a lot of promotion” (Navigator)
	**Logistic Barriers**
	“They don’t want to spend a ton of time going through things.” (Genetic counselor) “We have found that having people travel, even if we pay for travel, was a big barrier to service.” (Genetic counselor)
	**Emotions**
	“Removing that sense of fault. I see a lot of women who say “I cannot tell my family that this is going on” (…) “I can’t put this on them” like it is a burden they are putting on them” (Genetic counselor) “A lot of fear, fear of the results, of what the results will be, what will happen, so they don’t want to know if they are going to get cancer or not (…) fear of the costs” (MD, geneticist)
	**Spirituality**
	“I hear a lot of patients say you know it’s not the doctor, the doctors they can say what they want to say but it’s not them who have the last words, it’s God who has the last word with this.” (Psychology/counselor)
	**Fatalistic Beliefs**
	“I am not in control of the situation, so me doing a test is not going to make a difference” (Genetic counselor) “Whatever is going to happen, whatever God has in mind for me is what is going to happen. So, this idea that the information won’t necessarily change an outcome” (Genetic counselor)
	**Stigma**
	“I think there is stigma associated with it, especially back in those days, and especially in rural areas” (Navigator) “There was limited understanding about the causes of cancer (…) any disease that was considered contagious or that diminished the abilities in a family brought shame to the family ‘my family is unhealthy’” (Genetic counselor)
	**Systemic Barriers**
	“It is not only the obligations as a caretaker but also as the person who supports economically the family” (Psychologist).

**Table 2:** Experiences during Counseling.

**Family History**
“I’ve seen mainly women from El Salvador (…) someone had cancer and they didn’t know what type. Or it would be vague like, “they had cancer in their womb” but does that mean they had uterine cancer? Was that cervical cancer-was that ovarian cancer? It’s hard to know.” (Genetic counselor) “I’d say they know a little less about the family history. And they will communicate why. They’d say you know “with my family members it’s about culture: we don’t talk about illness,” or “when people died in my country, they just died. No one really talked about it much” or “a lot of people didn’t go to the doctor.” So on our family trees we have a lot of question marks regarding what could be the causes of death for people. Or occasionally it’s just cancer and we don’t have more specifics, which makes it hard to give a patient an accurate risk assessment.” (Genetic counselor)
**Communication with Relatives**
“That has been my biggest challenge. I have a few families where they have relatives coming afterward to be tested by me. So I don’t know how many of them are getting tested or who they end up communicating their test results to.” (Genetic counselor) “They don’t want to worry their family, so they haven’t told their families, their moms who live in a different country or they haven’t told their children or whatever it is. And I think that creates a lot a huge burden to keep that to yourself” (Genetic counselor)
**Testing Relatives Outside the US**
“I think that’s another thing that comes up a lot. They tell me, “that’s great that you tell me this information and that they need to do this testing or screening, but they don’t have access to it (…) so is it fair for me to do this testing and be like ‘good luck everyone else’?” (Genetic counselor) “So, we have a research registry with collaborating sites in Mexico and in Colombia and in Brazil (…) And sometimes those options are still very limited, especially when they live on the other side of the country. And so, it might not be feasible and then the other challenge, of course, is that if they test positive, they would not have access to those services that they will need.” (Genetic counselor)
**Language**
“Working through a translator is very difficult; the translation information isn’t really detailed and nuanced. So, it’s really a challenge for me to break down the information as much as I can to make sure that the patient is getting a good understanding of what we’re doing and why we’re doing it and that they’re able to make the same type of choice as someone who is an English speaker.” (Genetic counselor). “You take someone that doesn’t really have much health literacy or much anatomy, they are drawn into this world where people are throwing words at them (…) if it is in a language not even in your native language. And then you are trying to make decisions about these things when you can’t even grasp the most basic and what you even have. I imagine it is overwhelming.” (Genetic counselor)
**Emotions**
“Guilt, um blame, like you said if it’s they don’t have a good relationship with someone like their mother and then it turned out that it is maternally transmitted they blaming people who you don’t have a good relationship for this we’ve seen joy when you test negative which is almost I don’t wanna to say a full sense of security but having to kind of bring them down (…)” (Genetic counselor) “The parent is like ‘I didn’t even know that I had this (deleterious mutation). I’ve given it to my child and here my child is suffering because of something that I wasn’t even aware of.’ That’s where I see guilt the most I think. And then also too with young women who have been recently diagnosed and also have small children. It’s like, ‘not only am I sick,’ but there is this added pressure to like ‘taking care of my children but also now my children are at risk. I may not be there for them when they go through this too.” (Genetic counselor)
**Education and Misconceptions**
“Because one of them is the belief that all of us have cancer inside of us and it’s just waiting to be turned on and so this belief that everybody has cancer cells and those cancer cells are waiting to be activated (…) (Genetic counselor) “Gynecologic cancers can often be misreported, so while they say it’s ovarian, it was really uterus or cervical cancer (…) within Latinas and Spanish specifically (…) that is a common misunderstanding overall because some of the words they use interchangeably to describe female cancers.” (Genetic counselor)
**Engagement in Decision-Making (Respect)**
“When someone has a BRCA1 mutation, I would want them to have their ovaries removed too, but I want them to want to have their ovaries removed, to really understand why. And I think that their idea of what they look at as elective surgery is a hard thing to consider.” (Genetic counselor) “I like women to be engaged in discussing that and choosing what’s best for them, and what feels most comfortable. And I feel like maybe with the Latina women I see, it’s whatever I recommend” (Genetic counselor)
